# Socially Relevant Approaches to the Detection of Dementia in Minority Older Adults

**DOI:** 10.1093/geroni/igab046.2669

**Published:** 2021-12-17

**Authors:** Lana Sargent, Sarah Lageman, Leroy Thacker, Sally Russell, Marissa Mackiewicz, Elvin Price

**Affiliations:** 1 VCU School of Nursing, Ashland, Virginia, United States; 2 VCU Parkinson's & Movement Disorders Center, Richmond, Virginia, United States; 3 Virginia Commonwealth University Wright Center for Clinical and Translational Reserach, Richmond, Virginia, United States; 4 VCU School of Nursing, Richmond, Virginia, United States; 5 VCU Institute for Inclusion, Inquiry, and Innovation (iCubed): Health and Wellness in Aging Populations, Richmond, Virginia, United States; 6 Virginia Commonwealth University/ School of Pharmacy, Richmond, Virginia, United States

## Abstract

Substantial gaps remain in the scientific literature regarding low-income minority older adult populations with Alzheimer’s disease and related dementias (ADRDs). Access to care and early cognitive screening are often barriers to advancing ADRD detection in low socioeconomic status (SES) minority older adults. Additionally, there is the need for demographically (age, education, sex, race, ethnicity, and income) corrected normative scores in cognitive measures. Our cross-sectional study evaluated the psychometrics of the Mini-Mental State Exam-2 (MMSE-2) and the NIH Toolbox Cognition Battery (NIHTB-CB). The sample consisted of n=80 community-based older adults without a diagnosis of dementia living in low-income high-rise housing units. Acceptability is assessed with a brief 6-item acceptability survey, multiple linear regression is used to get predicted cognitive scores adjusted for age, education, income, ethnicity, race, and sex, and t-test comparison of the adjusted scores found in this study to established norms. Results found a mean age of 73, 70% black, 48% with < 12th-grade education, 51% have a monthly income of < $1,000, and 49% with undiagnosed cognitive impairment (CI) by both measures. When applying demographic adjustments in the NIHTB-CB 1) standard scores; 2) age-corrected scores, and 3) demographically correct scores all remained significant (p > 0.0001). Participants reported high (80-95%) acceptability for the community-based cognitive screening, 18% reported concerns with cultural appropriateness of the questions in the NIHTB-CB as compared to 5% with the MMSE-2. This research lays the foundation for a community-based cognitive screening and care coordination program for the low SES minority older adult population.

